# Probiotics, a promising therapy to reduce the recurrence of bacterial vaginosis in women? a systematic review and meta-analysis of randomized controlled trials

**DOI:** 10.3389/fnut.2022.938838

**Published:** 2022-09-20

**Authors:** Wei Keong Chieng, Muhammad Irfan Abdul Jalal, Jashveerdeep Singh Bedi, Ani Amelia Zainuddin, Mohd Helmy Mokhtar, Muhammad Azrai Abu, Kah Teik Chew, Abdul Ghani Nur Azurah

**Affiliations:** ^1^Department of Obstetrics and Gynecology, UKM Medical Centre, The National University of Malaysia, Kuala Lumpur, Malaysia; ^2^UKM Medical Molecular Biology Institute, UKM Medical Centre, The National University of Malaysia, Kuala Lumpur, Malaysia; ^3^Department of Obstetrics and Gynecology, Hospital Raja Perempuan Zainab II, Kota Bharu, Malaysia; ^4^Department of Physiology, Faculty of Medicine, The National University of Malaysia, Kuala Lumpur, Malaysia

**Keywords:** standard antibiotics regimen, 1-month postintervention, single menstrual cycle, probiotics effectiveness, risk ratio, meta-regression

## Abstract

**Introduction:**

The evidence for probiotic efficacy in preventing bacterial vaginosis (BV) recurrences among women aged 18 years and above is sparse. We aimed to ascertain the efficacy of probiotics in preventing BV recurrences after at least one menstrual cycle in this population.

**Methods:**

We conducted a systematic literature search using PubMed, MEDLINE (Ovid interface), Web of Science (WoS), Scopus, Embase, ProQuest Dissertations and Theses Global, Cochrane Library databases and registries comprised of Open Science Framework (OSF) preprints registry, the ClinicalTrials.gov (USA), WHO International Clinical Trials Registry Platform (WHO-ICTRP), International Standard RCT Number (ISRCTN) registry, limited to randomized clinical trials (RCTs) in English published between January 2000 and December 2021. The inclusion criteria were trials that administered probiotics to BV-positive women in an experimental arm of at least 20 samples. The usage of probiotics should be preceded with standard antibiotic regimen and followed by a reassessment of BV status after at least a single menstrual cycle. Risk of bias assessment was completed using revised Cochrane risk-of-bias tool for randomized trials (RoB 2). The PROSPERO registration number of the review is CRD42022302044.

**Results:**

From 8,162 identified records, we included 10 studies (*n* = 1,234 participants) for final analysis; 7 trials compared probiotics vs. placebo, whereas 3 trials compared probiotics vs. metronidazole alone. Using random-effects meta-analysis, probiotics were shown to reduce the risk of BV recurrences by 45% compared to either placebo or metronidazole [14.8 vs. 25.5%, RR: 0.55 (95%CI: 0.33, 0.91), *p* = 0.03, *I*^2^ = 45.4% (95%CI: 0, 73.7%)]. Sensitivity analysis revealed the robustness of results upon removal of studies with high risk of bias [RR: 0.54 (95%CI: 0.38, 0.77), *p* = 0.006] and reporting bias (RR: 0.53, 95%CI: 0.39, 0.74, *p* = 0.002). Meta-regression demonstrated that the route of administration (*p*_vaginal_ = 0.67; *p*_oral_ = 0.44), the total dosage of probiotics (*p* = 0.17), cumulative days of probiotic administration (*p* = 0.76), and the number of species in probiotic preparation (*p* = 0.40) were not linked to BV recurrences.

**Interpretation:**

Probiotics were associated with more than twofold reduction in BV recurrences when BV status was assessed after at least 1-month postintervention. Further high-quality and methodologically standardized RCTs should evaluate probiotic efficacy for BV prevention in a diverse community setting.

**Systematic review registration:**

[https://www.crd.york.ac.uk/prospero/display_record.php?ID=CRD42021290613], identifier [CRD42021290613].

## Introduction

While the medical field is advancing, bacterial vaginosis (BV) remains a nuisance to the reproductive health of women. The BV is characterized by an imbalance of the normally *Lactobacilli*-dominated vaginal microbiome. This condition sets in when the overgrowth of potentially pathogenic bacteria, such as the frequently studied *Gardnerella vaginalis*, *Atopobium vaginae*, *Mobiluncus* sp., and *Mycoplasma hominis* colonize the vaginal ecosystem (1). Being the most common cause of vaginal symptoms among women of reproductive age, it is not surprising that BV has a high prevalence which ranges from 23 to 29% across regions globally that lead to an estimated annual global financial burden of USD 4.8 billion for symptomatic BV treatments (2). Although BV can be asymptomatic, approximately 50% of women experience symptoms like vaginal malodor, itching, and discharge (3). These disturbing symptoms have certainly decreased their quality of life, especially when BV recurrences occur (4, 5). BV is also known to cause multiple obstetrical and gynecological complications, which include but not limited to preterm birth, pelvic inflammatory disease, cervical intraepithelial neoplasia, and increased susceptibility to infections such as the acquisition of human immunodeficiency virus and human papillomavirus (6). Recently, it has even been associated with infertility and potential impedance on the success of assisted reproductive therapy (7).

Despite BV’s costly impacts at individual and societal levels, to date, there is no effective treatment for BV recurrences. Current standard BV treatments remain antibiotics, namely metronidazole and clindamycin. The cure rate of BV in studies using antibiotics varies greatly between 46.75 and 93.86%, depending on the definition of cure and study protocol of respective study (8). Metronidazole and clindamycin were reported to be equally efficacious, regardless of route of administration when compared within the same intervals (9). Numerous studies have reported a high recurrence rate of BV, which varies from 23% at 1 month to 50–60% by 12 months, despite an initial effective treatment of BV (10–12). Apart from the known side effects of antibiotics, its usage is undermined by potential antibiotic resistance, their potential eradicating effects of healthy vaginal microbes, and inability to restore the *Lactobacilli*-dominated normal vaginal flora. Although the idea of vaginal microbiota transplant has gained prominence as a strategy for vaginal ecosystem restoration in cases of BV recurrences (13), the less invasive alternatives should be prioritized. A lot of efforts are being put into the discovery of agents that do not only treat but also prevent BV recurrences. Probiotics containing *Lactobacilli* appear to be one of the promising agents.

According to the Food and Agriculture Organization of the United Nations (FAO) and the World Health Organization (WHO), probiotics are defined as live microorganisms that when administered in adequate amounts, confer a health benefit on the host (14). In a healthy vaginal ecosystem, species from the Lactobacillaceae was found to be the predominant species (15). It is thought that, through symbiotic relationship between *Lactobacilli*, host’s vaginal microbiota and immune system, colonies of pathogenic and opportunistic microbiota such as *Candida* and *Gardnerella vaginalis* can be kept under control through the inhibition of biofilm formation, nutritional competition in the vaginal microenvironment, and synthesis of antimicrobial substances such as lactic and acetic acid and hydrogen peroxide that impede pathogenic microbiota proliferation as well as immune system modulation through the maintenance of interleukin 4 (IL-4) and IL-10 production (15–17). Hence, the restoration of normal vaginal microflora through *Lactobacilli* supplementation is considered to be a viable adjunctive strategy for the treatment or prevention of BV (18).

This study aims to determine the efficacy of probiotics in reducing BV recurrences after at least a menstrual cycle and ascertain whether factors such as route of administration, total dosage of probiotics, number of strains, cumulative days of probiotic consumption, administration of *Lacticaseibacillus rhamnosus* strain, individual study’s risk of bias, sample size, impact factor, and publication year influence the efficacy.

## Methods

### Study protocol and research question

This study was registered in the International Prospective Register of Systematic Reviews (PROSPERO) (Protocol number #CRD42022302044) and conducted in accordance with the Preferred Reporting Items for Systematic Reviews and Meta-Analysis (PRISMA) 2020 guidelines (19). The research question of this review was framed based on the PICOS framework: Population (P): Premenopausal female aged 18 and above with BV; Intervention (I): Probiotics (either oral or vaginal route); Control (C): Placebo or metronidazole alone; Outcome (O): BV recurrence rate; Study Design (S): Randomized Controlled Trials (RCTs).

### Data sources, search strategy, and eligibility criteria

Systematic searches were conducted in electronic databases which included PubMed, MEDLINE (Ovid interface), Web of Science (WoS), Scopus, EMBASE, ProQuest Dissertations and Theses Global, and Cochrane Library. Manual searching of the references in the relevant published articles was also performed. For gray literature detailing the preventive effects of probiotics and BV, customized Google search was conducted. Searches were also done in Open Science Framework (OSF) preprints registry and the controlled trial registries, which encompassed the ClinicalTrials.gov (USA), WHO International Clinical Trials Registry Platform (WHO-ICTRP), and International Standard RCT Number (ISRCTN) registry to find additional gray literature. Nine corresponding authors were contacted to either acquire unpublished study results of published trial protocols relevant to our study, or to clarify information in original manuscripts. Despite following up, only 2 authors responded to our enquiries.

The Medical Subject Heading (MeSH) terms: (1) “Vaginosis, Bacterial”; (2) “Probiotics”; (3) “*Lactobacillus*”; (4) “Recurrence”; and (5) “Prevention and control” were combined using BOOLEAN operators (AND/OR) as [1 AND (2 OR 3 OR 4 OR 5)] for the searches.

The eligibility criteria for studies considered for inclusion were those of RCT design, fulfilled the operational definition of recurrence (verification of cured BV followed by BV reappearance after at least one menstrual cycle), at least 20 samples in each of the intervention and placebo arms, and published in English between January 2000 and 27th December 2021 with full-text available. The studied population was therefore premenopausal, non-pregnant, and non-lactating women with BV diagnosed using either the clinical Amsel’s criteria (3 out of 4 criteria) or the Nugent Gram Stain (NGS) scoring system (score of 7–10) at first medical contact. They were subsequently cured with standard BV antibiotic regimen. The cure was confirmed by the non-fulfillment of BV-positive status (i.e., Amsel < 3 criteria or Nugent score < 7). They then received probiotics (for the treatment arm) before being followed up after at least a single menstrual cycle to detect the presence of recurrences.

The exclusion criteria of studies are the use of prebiotics, probiotic preparations in the form of food intake (e.g., yogurts), pregnant women, or women with HIV. Research publications from the same authors or institution were scrutinized to eliminate any data redundancy. In the case of redundancy, only results from the most recent publication were included.

### Outcomes

The primary outcome was pooled risk ratio on the recurrences of BV after probiotic administration. Meta-regression based on characteristics of studies such as route of administration, total dosage of probiotics, number of strains, cumulative days of probiotic consumption, administration of *Lacticaseibacillus rhamnosus* strain, risk of bias, sample size, impact factor, and publication year were also carried out since k ≥ 10. All tests were two-tailed, and *p*-values of 0.05 or lower were considered to be statistically significant.

### Data collection and risk of bias assessment

Two independent reviewers (WC and JB) identified the studies extracted from the searches. The filters on PubMed and MEDLINE (Ovid interface) databases were set to limit the search results to studies published in English and of RCT design. In other databases without automation tools to filter out non-RCT studies, manual exclusion was done by the reviewers. The results were imported into the EndNote version 20 library. Duplications were identified and removed. Titles, abstracts, and keywords of each retrieved record were screened to exclude any papers not fulfilling inclusion criteria. When there was uncertainty on the eligibility of an article, the study was adjudicated based on the discussion and consensus between the two reviewers and a third reviewer (AG). All excluded records were given reasons for exclusion.

The eligible studies were exported to Microsoft Excel 16.56. The Excel data extraction form recorded the following information: author/s, study title, study design, year of publication, DOI, population, intervention, comparator, route of administration, duration (interval) of follow-up, primary results, secondary results, types and number of species from the *Lactobacillaceae* family used, total dosage of probiotics received prior to reassessment for recurrences, adverse effects, and risk of bias. The standard Excel format (.xslx) was then converted to.csv or.txt format to ensure R recognized the data format and for easier analytical implementation on R.

The revised Cochrane risk-of-bias tool for randomized trials (RoB 2) was utilized to assess the risk of bias (20), and the Grading of Recommendations, Assessment, Development and Evaluation (GRADE) criteria was used to evaluate the quality of corpus of evidence retrieved for the study outcome (21). The assessment was conducted independently by two reviewers (WC and IJ). The risk of bias was assessed on the basis of intention-to-treat, according to the five domains of the tool: (1) Randomization process, (2) deviations from the intended interventions, (3) missing outcome data, (4) measurement of outcome, and (5) selection of the reported result. The reviewers then cross-checked the results of the assessment. Should there be any discrepancies in opinions, a third independent reviewer (AG) addressed the differences in opinion and reached a consensus together with the two reviewers.

### Operational definition

The recurrence of BV was defined as the reoccurrence of one or more episodes of BV after the completion of an episodic regimen (12). For systemic intervention that was administered for a longer period of time and/or has a longer half-life, the FDA recommended the verification of BV cure to be conducted within 21–30 days after randomization (22). For the purpose of this review, the definitions were further adapted. Thus, the recurrence of BV was operationally defined as reappearance of BV after at least a single menstrual cycle (28 days), counted from the day of clinical verification of cured BV. A non-BV status classified using either the Amsel’s criteria or Nugent score was considered cured. For studies that followed up patients for a duration greater than 1 month from the day of BV cure verification, the next immediate timepoint that was at least 1-month long will be used as reference point to determine BV recurrence (e.g., in a study that followed up patients on monthly basis for 6-month duration, if verification of cure was on month 2, month 3 would be reference timepoint in which cases of recurrence on month 3 in relation to month 2 would be captured for data analysis).

The route of administration of probiotics was coded as either oral or vaginal. The oral preparations include capsule, tablet, and sachet, whereas the vaginally delivered probiotics existed in the form of either capsule, pessary, or gel. The probiotics could be of single or mixed strains with any dosage.

### Statistical analysis

Meta-analysis was performed using the R packages, meta-version 3.0-2 (23), meta-version 5.2-0 (24), dmetar version 0.0.9000 (25), and metasens version 1.0-1 (26). It was implemented in R version 4.1.1 (27) on the RStudio version 1.4.1717 interface (28).

Using a random-effects model, the pooled risk ratios for rate of BV recurrences in the experimental and control group were computed using the Mantel–Haenszel method (without continuity correction) for weight assignment. The Paule–Mandel (PM) estimator was used to estimate the between-study heterogeneity variance, the Hartung–Knapp–Sidik–Jonkman method for the confidence intervals of summary effects, and the Q-profile method for 95% CI of tau^2^. Prediction interval (PI) was constructed to assess the magnitudes of effects of future studies in light of the current evidence.

Between-study heterogeneity was assessed using the Cochran χ^2^-test (*p* < 0.1 indicates significant heterogeneity), and the conventionally classified (0–40%: possibly unimportant; 30–60%: moderate heterogeneity; 50–90%: substantial heterogeneity; 75–100%: considerable heterogeneity) *I*^2^ statistic.

Sensitivity analysis was conducted by testing the robustness of the obtained effect measures (risk ratios) upon removal of: i) open label trials; ii) trials that used metronidazole alone; iii) trials that had high risk of bias and improper randomization; and iv) trials that exhibited high risk of selective outcome reporting bias.

Publication bias was evaluated by visual inspection of the funnel plots and objectively by Egger’s test. A significant *p*-value for the intercept term of Egger’s regression indicated the presence of publication bias.

Using simple meta-regression, we screened and investigated the influence of multiple summary-level variables [study characteristics and reporting status (high risk of bias, sample size, impact factor of journal where the report was published, and year of publication), the probiotics’ route of administration, total dosage of probiotics received, cumulative days of probiotic administration, and the number of species in probiotic preparations) on BV recurrences. Following that, multiple meta-regression models regressing relative risk of BV recurrences against total dosage of probiotics received; and risk ratio against total dosage of probiotics and route of administration were built. The ANOVA test was used to identify the best model. Permutation test was further undertaken to assess the robustness of the final selected model. All tests were two-tailed, and *p*-values of 0.05 or lower were considered to be statistically significant.

## Results

### Systematic search

**FIGURE 1:**
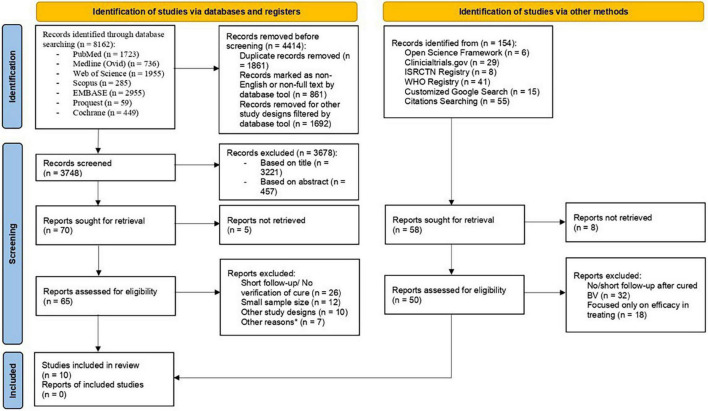
The PRISMA flow diagram summarizing study selection process.

The systematic searches of electronic databases yielded 8,162 articles, whereas searches in registries and manual searching of references had no additional eligible articles identified. There were 4,414 articles removed prior to screening, with the remaining 3,748 screened based on title and abstract. Retrieval of 70 articles returned 65 full-text articles with 5 not being able to retrieve. Ultimately, 10 RCTs were included in this review (29–38). Supplementary material 1 showed excluded studies with reasons. Details on the selection process of articles were represented with a flow diagram (Figure 1). In total, 40% of the studies had low risk of bias, whereas the number of studies with some concerns and high risk of bias were equal in number. There were 2 trials that exhibited discrepancy from their initial plan of analysis. The risk of bias assessment on included studies were available in Supplementary material 2.

### Study characteristics

Out of the 10 studies, 1 was designed as an open-label trial; 2 were single-blinded with the rest being double-blinded. A total of 1,234 premenopausal women (682 in control arm and 552 in intervention arm) in the 10 trials fulfilled the review’s operational definition of BV recurrence, with the sample size ranged from 58 to 268. All the women were treated with standard BV antibiotic regimen (7 used metronidazole; 1 used clindamycin), except in 2 studies in which the treatment prior to verification of cure was not specified. There were 7 studies that used placebo in the comparator group; 3 studies that gave metronidazole alone to women of the control group. Thirty percent (30%) of studies administered probiotics orally (33.33% sachet and 66.67% capsules); with the remaining 70% of studies administered probiotics vaginally *via* either capsules, pessary, gel, or tablets. The daily dosage of probiotics ranged from 1.0 to 5.4 billion CFU for the oral route and 40,000–8.0 billion CFU for the vaginal application. For the oral probiotic consumers, the highest frequency of consumption was twice per day, in contrast to vaginal probiotic users that had the highest frequency of only once per day. Overall, the cumulative duration of probiotic usage prior to reassessment for BV recurrences ranged from 8 to 60 days. The strains of probiotics used included *Lacticaseibacillus rhamnosus*, *Limosilactobacillus reuteri*, *Lactobacillus crispatus*, *Levilactobacillus brevis*, *Lactobacillus acidophilus*, *Streptococcus thermophilus*, and *Lactobacillus gasseri*. In total, 50% of the studies used only single strain, whereas another half used at least 2 different strains in their probiotic preparations. Further details on characteristics of studies are as shown in Supplementary material 3.

### Safety profile of probiotics

Among the 10 studies, only 8 had looked into adverse effects of probiotic administration. Among them, the common local side effects were vaginal itching and vaginal discharge, whereas the commonly reported unfavorable systemic effects were abdominal discomfort and frequent urination. Two trials reported adverse events attributable to the consumption of probiotics: 1/48 (2.1%) had an allergy reaction toward probiotics, and 1/98 (1.0%) was unable to tolerate oral consumption. There were three trials that did not comment on the relationship between the adverse events and probiotic usage; meanwhile, two trials revealed no consistent relationship between the two. There was one trial that reported no side effects at all.

### Probiotics vs. placebo/active treatment for prophylaxis of bacterial vaginosis recurrences

**FIGURE 2:**
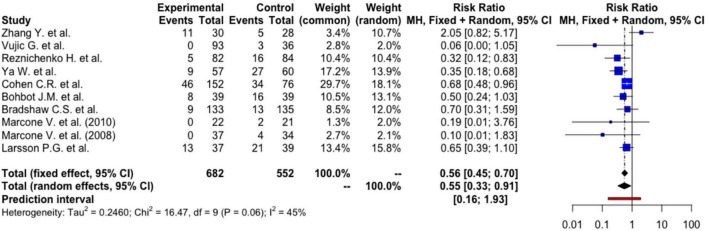
Forest plot on risk ratio of BV recurrence.

Using a random-effects model, women who had received probiotics had a 45% lower risk of BV recurrences [RR: 0.55 (95%CI: 0.33, 0.91), *p* = 0.026] (Figure 2). The absolute risk reduction is 12.12% [95% CI: 2.18%, 17.06%] with a number needed to treat (NNT) of 8.25 [95% CI: 5.86, 45.85]. This means that we need to treat 9 patients to prevent one BV recurrence, and this can be as few as 6 patients or as many as 46 patients at the population level.

The between-study heterogeneity variance was estimated at tau^2^ = 0.25 [95%CI: 0, 2.43], with an *I*^2^-value of 45.4% [95%CI: 0, 73.7%]. The PI ranged from *g* = 0.16 to 1.93, indicating future studies may exhibit a mixture of positive and negative treatment effects. The broad interval suggested high effects may be possible as well. Although an *I*^2^-value lesser than 50% was conventionally regarded as more homogenous, further analyses were based on random-effects model to take into account factors such as differences in methodology, inclusion criteria, dosage of probiotics, duration of administration, and strains of probiotics that may contribute to heterogeneity. Meta-regression was also performed to identify any sources of heterogeneity.

### Outlier identification and influential analysis

**FIGURE 3:**
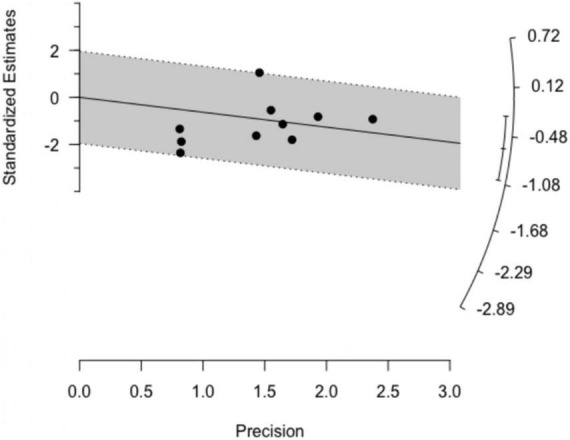
The Galbraith plot showed no presence of outliers.

Both basic outlier removal and the Galbraith plot (Figure 3) revealed no outlier among the included studies. Influential analysis found none of the studies exerted high influence on the overall results (Supplementary material 4), thereby increasing the robustness of our findings. These results were further supported by a rather symmetrical, homogenous distribution in the GOSH plot. A diagnostic GOSH diagram is available as Supplementary material 5.

### Sensitivity analyses

The effect of inclusion of studies with high risk of bias in the meta-analysis to the effect measures was examined using sensitivity analyses. A sensitivity analysis that excluded 3 studies (*k*_sens–bias_ = 7) with high risk of bias (non-double-blinded studies, use of active treatment instead of placebo in the control group, and insufficient randomization) did not affect the certainty of our initial meta-analysis [RR: 0.54 (95%CI: 0.38, 0.77), *p* = 0.006] with similar PI (*g* = 0.30–1.00). The sensitivity analysis that excluded 1 open-label trial (*k*_sens–open_ = 9) also had similar results [RR: 0.53 (95%CI: 0.39, 0.74), *p* = 0.002], with a similar PI (*g* = 0.32–0.88). The sensitivity analysis of 8 studies (*k*_sens–report_ = 8) after exclusion of the 2 studies with high risk in the selection of reported results domain revealed a comparable result [RR: 0.53 (95%CI: 0.37, 0.76), *p* = 0.004] and a comparable PI (*g* = 0.28–0.99).

### Publication bias

**FIGURE 4:**
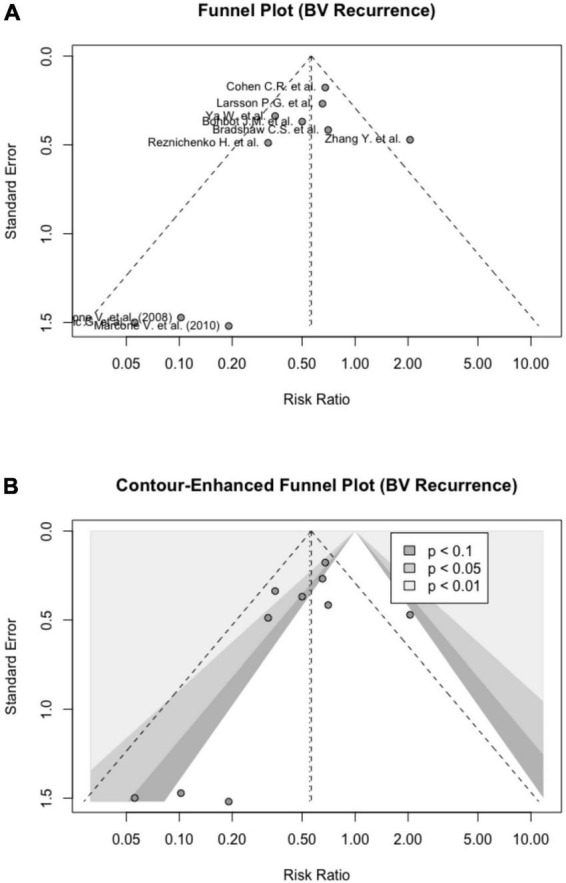
Visual inspection of publication bias. **(A)** Funnel plot; **(B)** contour-enhanced funnel plot.

The publication bias was not detected based on the symmetrical Funnel plot and a non-significant Egger’s test [intercept = − 1.082 (95% CI: − 2.58, − 0.42), *p* = 0.195] (Figure 4A). Figure 4B shows a contour-enhanced Funnel plot. Based on Rücker’s meta-analysis limit method, probiotics were still effective in reducing BV recurrences even after adjusting for small study effects (*g* = 0.74) (Supplementary material 6).

### Grading of recommendations, assessment, development and evaluation

Initially, the quality of the corpus of evidence for BV recurrences was apparently high since all studies included are RCTs. The evidence was downgraded at one point for risk of bias due to the lack of or insufficient reporting on allocation concealment in most of the trials included in this review. Overall, the quality of evidence demonstrating the efficacy of probiotics in preventing BV recurrences was moderate (Table 1).

**TABLE 1:** GRADE assessment on the quality of the evidence reviewed for BV recurrences.

Quality of evidence							Evidential summaries				
			
Total number of participants (no of RCTs)	Risk of bias	Inconsistency	Indirectness	Imprecision	Publication bias	Overall judgment on quality of evidence	Event rates (%)		Relative risk (random-effects model)	Risk with placebo/ antibiotics	Absolute risk difference with probiotics (95% CI)
		
							Probiotics	Placebo/ antibiotics			
1234 (10 RCTs)	Serious^b^	Not serious	Not serious	Not serious^a^	Not serious	Moderate	101/682 (14.8%)	141/552 (25.5%)	0.55 [95% CI: 0.33, 0.91]	255 per 1,000 population	12.12% (5.86, 45.85)

^a^The calculated Optimal Information Size (OIS) is 698 at 80% power, 5% type I error rate, *p*_control_ = 0.2554, and relative risk = 0.75 (corresponding to 25% relative risk reduction). ^b^Lack of allocation concealment in half of the trials.

### Meta-regression

The mixed-effects meta-regression of risk ratio (*k* = 10) showed that risk of bias [high risk: β = 0.16 (95%CI: − 1.18, 1.50), *p* = 0.786; some concerns: β = − 0.65 (95% CI: − 2.16, 0.87), *p* = 0.345; low risk: β = − 0.94 (95%CI: − 2.43, 0.55), *p* = 0.181], sample size [β = − 0.0001 (95%CI: − 0.0072, 0.0069), *p* = 0.970], impact factor of journals [β = 0.0031 (95%CI: − 0.0121, 0.0184), *p* = 0.648], and publication year of articles [β = 0.04, (95%CI: − 0.05, 0.14), *p* = 0.341] were not significant moderators. These suggested that the inclusion of studies with high risk of bias in the analysis did not affect our effect measures. Furthermore, the route of administration (oral or vaginal) was not a significant predictor for BV recurrences [vaginal: β = − 0.24, (95%CI: − 1.47, 1.00), *p* = 0.672; oral: β = − 0.39, (95%CI: − 1.49, 0.71), *p* = 0.443]. Similarly, the total dosage of probiotics received [β = 0 (95%CI: 0, 0), *p* = 0.174], cumulative days of probiotic consumption [β = − 0.0043 (95%CI: − 0.0356, 0.0269), *p* = 0.757], and the number of species in the probiotic preparations [β = − 0.20 (95%CI: − 0.78, 0.39), *p* = 0.460] were not predictive of BV recurrences. The presence of *Lacticaseibacillus rhamnosus* strain in the probiotics administered was also a not significant confounding factor for the risk of BV recurrences [β = 0.02 (95%CI: − 1.04, 1.09), *p* = 0.962].

In a multiple regression model consisting of total dosage received prior to reassessment of BV recurrences and route of administration, the Omnibus test was not significant (*p* = 0.093) although the total dosage was a significant predictor [β = 0 (95%CI: 0, 0), *p* = 0.039] within the model. With a permutation test of the same model, the Omnibus decreased from the initial *p*-value of 0.093–0.061. The meta-regressions are summarized in Table 2, with bubble plots available in Supplementary material 7.

**TABLE 2:** Mixed-effects meta-regression of risk ratio on characteristics of study.

Covariates	Coefficient	95% CI	Standard error	*P*-value
**Risk of bias**				
• High • Some concerns • Low	0.1603 –0.6492 –0.9370	[–1.1806, 1.5012] [–2.1640, 0.8657] [–2.4283, 0.5543]	0.5671 0.6406 0.6307	0.786 0.345 0.181
Sample size	–0.0001	[–0.0072, 0.0069]	0.0031	0.970
Impact factor	0.0031	[–0.0121, 0.0184]	0.0066	0.648
Publication year	0.0419	[–0.0536, 0.1375]	0.0414	0.341
**Route**				
• Vaginal • Oral	–0.2357 –0.3851	[–1.4729, 1.0014] [–1.4851, 0.7148]	0.5365 0.4770	0.672 0.443
Total dosage received	0.0000	[0.0000, 0.0000]	0.0000	0.174
Cumulative days of probiotic consumption	–0.0043	[–0.0356, 0.0269]	0.0136	0.757
Number of species	–0.1957	[–0.7767, 0.3854]	0.2520	0.460
Presence of *Lacticaseibacillus rhamnosus*	0.0228	[–1.0393, 1.0850]	0.4606	0.962

## Discussion

Probiotics is an efficacious prophylactic agent to prevent post-treatment BV recurrences at intervals of at least 1 month, regardless of route of administration. Numerous studies had demonstrated improvement in the BV cure rate after probiotic administration (39–42). Research on the prevention of BV recurrences using probiotic supplementation was critically inadequate. Consistent with our observations, 3 recent systematic reviews and meta-analyses (41, 43, 44) had demonstrated the efficacy of probiotics in preventing BV recurrences at 1- to 3-month intervals. Previous narrative review (42) also revealed the promising potential of probiotics as a prophylactic agent against BV relapses. However, those systematic reviews searched a limited number of databases, included individual trials with small sample size and lacked standardized operational definition of BV recurrence and clarity in reporting (e.g., the number of studies analyzed for each of the subgroup analyses and statistical methods used to control the effects of moderating variables [confounders] were not elucidated). In contrast to positive findings of the aforementioned research, some authors reported that probiotics did not increase BV cure rate (29), did not exhibit strong positive effects (45), and had little significant effects in the treatment of BV when added to antibiotic regimen (46). These stark differences in research findings can be attributed to the differences in study design across studies, such as the ethnicity of study samples, species of probiotics, and dosage of probiotics used. Each strain exhibits different properties in modulating the vaginal microbiome. For instance, previous study (47) demonstrated that *Lactobacillus crispatus* produced higher level of lactic acid compared to *Lactobacillus iners*, *Lactobacillus gasseri*, and *Lactobacillus jensenii*. Moreover, the same study also showed women of different ethnicity had differential vaginal pH levels, which were lowest in White, followed by Asian, Black, and Hispanic study subjects. These findings thus suggested that different populations may have incongruous probiotic efficacy against BV.

Surprisingly, in our studies, none of the hypothesized confounders that may moderate the efficacy of probiotics, such as route of administration, total dosage of probiotics received, duration, and number of species in probiotics, were significant independent factors for BV recurrences. Nevertheless, the results should be interpreted cautiously as the number of studies included in the meta-regression analysis was rather limited (*k* = 10). Similar to our results, a probiotic administration for a 10-day duration resulted in a significant reduction of pathogenic microbiota in vagina and the maintenance of vaginal eubiosis up to 30 days after the end of treatment, regardless of the route of administration (48). In contrast, a recent systematic review found that *Lacticaseibacillus rhamnosus* administered orally was more efficacious than intravaginal application for the treatment of BV (49), which was again likely to be due to unstandardized methodology mentioned above. A narrative review had concluded that the administration of selective probiotic strains resulted in enhanced host’s ability to fight existing infection, restoration of normal vaginal flora and prevention of further BV recurrence when given at a dosage of over 100 million CFU for 2 months (50). However, the claim was debatable as trials using different dosage and duration of probiotic administration coherently showed similar results. The disparity in study results could not be established until a standardized study protocol is adopted universally, e.g., studies using the same population and the same dosage but different strains to determine the *in vivo* efficacy of strains, or studies using the same population and the same dosage but different duration of administration to ascertain a minimal duration that best prevents BV recurrences.

Despite the contradictory evidence on the efficacy of probiotics against BV, several mechanisms had been proposed for the modulatory effects of probiotics on microbiota. The *Lactobacilli* prevent the displacement of normal vaginal microbiome with pathogens by forming a mechanical barrier; compete functionally for receptors in mucosa and epithelium and for nutrition with pathogens thereby preventing colonization as a result of displacement and exclusion competition; directly interact *via* lectin-like adhesion components to block the adhesion of pathogens to epithelial cells; promote the immunomodulation mechanisms by provoking innate immunity system and stimulate anti-inflammatory mechanisms; and produce antimicrobial substances such as bacteriocins, lactic acid, and hydrogen peroxide (51, 52). The bacteriocins are heterogeneous chemicals that inhibit the activities of pathogens thereby removing them from the microbiome. Meanwhile, the hydrogen peroxide and lactic acid both maintain the physiological vaginal pH of 4.5 or less, hampering the growth of pathogens. The lactic acid neutralizes the electrochemical potential of cell membranes and intracellular protein denaturation of harmful microflora, protecting the epithelial cells against injury (52), whereas the hydrogen peroxide has the ability to kill the pathogens (53). Most importantly, *Lactobacilli* were found to inhibit biofilm formation of *Gardnerella vaginalis* (54), and restore and maintain normal vaginal flora, which help to treat existing infection and prevent recurrence of BV (50). A recent pilot clinical study that investigated the probiotic potential of *Lacticaseibacillus rhamnosus* had exhibited the strain to have broad adverse activities against vaginal pathogens, *in vitro* capability to adhere to VK2/E67 vaginal epithelial cells, and antioxidant properties as demonstrated by the strain’s ability to resist linoleic acid peroxidation. Furthermore, the strain was also shown to have anti-inflammatory properties in which gene expressions of COX-2 and the pro-inflammatory interleukin-8 were downregulated, whereas the expression of the anti-inflammatory interleukin-10 was upregulated (49).

To the best of our knowledge, this is the first meta-analysis and meta-regression on BV recurrence prophylaxis that attempted to mitigate the risk of publication bias by screening vast number of databases and Gray literature searching. The stringent inclusion criteria that required verification of clinically cured BV and at least an interval of 1-month prior to reassessment were opted to minimize between-study heterogeneity. Furthermore, our study is the first study employing the GRADE evaluation tool to assess the quality of evidence reviewed in our study. The concluded moderate quality of evidence in our review may assure and justify further pragmatic prospective RCTs at a community-level setting to ascertain the identified positive effects of probiotics on BV recurrences. This will enable probiotics to be one of the treatments in BV prophylaxis arsenal after initial cure using standard antibiotic regimen.

Our study, however, was hampered by several limitations. First, although longer interval of follow-up was preferred in determining the longest possible period that the women can be BV-free after probiotic administration (i.e., without BV recurrences), a majority of the trials did not follow up patients for more than 1-month period after verification of BV-free status. Second, we were unable to control the flaw in methodology such as insufficient blinding, inadequate allocation concealment for most trials, and possible substandard randomization. Third, unstandardized methodology between the trials such as self-sampling vs. clinician sampling, and treatment-related factors such as varied dosage of probiotics, duration of treatment, preparation forms, and species of probiotics, which may render the synthesized evidence to be less convincing. Fourth, we only included studies published in English, which may result in selection bias. Fifth, one of the studies demonstrated an opposite effect of probiotics on BV recurrences [RR: 2.05 (95% CI: 0.82, 5.17)], and this might potentially result in a further downgrading of the inconsistency domain of the GRADE assessment tool. However, since the lower boundary of its 95% CI for the relative risk of BV recurrences overlapped with the majority of other trials’ 95% CIs, we did not rate down the inconsistency domain of the GRADE assessment tool, a decision that is congruent with the recommendations made by the original GRADE working group (55).

This study added to the body of evidence by demonstrating the efficacy of probiotics in preventing BV recurrences even after an interval of 1 month. The meta-regression had also undoubtedly laid preliminary results that served as a foundation for future studies to determine the influence of confounding factors, such as route of administration, dosage, and duration on the efficacy of probiotics. Although the effects of probiotics in preventing recurrences were plausible, further high-quality evidence is required to confirm our findings. The moderating effects can only be robustly investigated with the availability of more evidence. Future trials should be strategized to reduce drop-outs while adopting a longer period of follow-up, preferably up to 1 year after the completion of probiotic treatment. Furthermore, future systematic review should also focus on inclusion of RCTs with larger sample sizes, preferably RCTs whose sample sizes were calculated *a priori* using statistical power analysis (56).

## Conclusion

The review showed that probiotics were efficacious in preventing recurrences of BV after initial cure by standard antibiotic regimen. Probiotics were found to almost halve the risk of BV recurrences when reassessment was done at the interval of at least 1 month. Multiple moderating factors did not demonstrate any influence on the efficacy of probiotics in preventing BV recurrences. Future studies with longer periods of follow-up and standardized design will be able to consolidate the current evidence.

## Data availability statement

The original contributions presented in this study are included in the article/Supplementary material, further inquiries can be directed to the corresponding author/s.

## Author contributions

WC, JB, MA, and AN conceptualized and designed the review, involved in the conduction of systematic searches. AZ and MM scrutinized the methodology of the study. WC and MA contributed to bias and quality assessment and performed the data analysis. MAA and KC validated the extracted data. WC, JB, MA, and AZ drafted the manuscript. AN, MM, MAA, and KC reviewed and edited the manuscript. AN provided close supervision to the study progress. All authors contributed to the article and approved the submitted version.
