# Supplementary material for: Probiotics, a promising therapy to reduce the recurrence of bacterial vaginosis in women? a systematic review and meta-analysis of randomized controlled trials
Source: Front Nutr. 2022 Sep 20;9:938838. doi: 10.3389/fnut.2022.938838 (PMC9530327; doi:10.3389/fnut.2022.938838)
Supplement: Supplementary file 1 [file Data_Sheet_1.docx]

**Supplementary Material 1:** List of excluded trials with reasons (n=55)

| **No** | **Trial references** | **Reasons for exclusion** |
| --- | --- | --- |
| 1. | Rostok M, Hütt P, Rööp T, Smidt I, Štšepetova J, Salumets A, Mändar R. Potential vaginal probiotics: safety, tolerability and preliminary effectiveness. Benef Microbes. 2019;10(4):385-393. doi: 10.3920/BM2016.0123. | Did not fulfill the review's inclusion criteria of following up at least after a single menstrual cycle upon cure of BV(total duration of study was 6 weeks, however, there was no information about verification of cure, thus recurrence) |
| 2. | Sgibnev A, Kremleva E. Probiotics in addition to metronidazole for treatment Trichomonas vaginalis in the presence of BV: a randomized, placebo-controlled, double-blind study. Eur J Clin Microbiol Infect Dis. 2020;39(2):345-351. doi: 10.1007/s10096-019-03731-8. | Did not fulfill the review's inclusion criteria of following up at least after a single menstrual cycle (duration of entire trial was 15 days only, inclusive of follow-ups) |
| 3. | Tomusiak A, Strus M, Heczko PB, Adamski P, Stefański G, Mikołajczyk-Cichońska A, Suda-Szczurek M. Efficacy and safety of a vaginal medicinal product containing three strains of probiotic bacteria: a multicenter, randomized, double-blind, and placebo-controlled trial. Drug Des Devel Ther. 2015;9:5345-54. doi: 10.2147/DDDT.S89214. | Did not fulfill the review's inclusion criteria of following up at least after a single menstrual cycle (whole duration of trial was 19 days, inclusive of 2 follow ups), samples were of intermediate Nugent score (4-6) |
| 4. | Ngugi BM, Hemmerling A, Bukusi EA, Kikuvi G, Gikunju J, Shiboski S, Fredricks DN, Cohen CR. Effects of bacterial vaginosis-associated bacteria and sexual intercourse on vaginal colonization with the probiotic Lactobacillus crispatus CTV-05. Sex Transm Dis. 2011;38(11):1020-7. doi: 10.1097/OLQ.0b013e3182267ac4. | Although vaginal samples were taken at day of enrollmenet and D28 of the trial, no BV status (cured/failed to cure) at these time points were reported as recurrence is not an area of interest to this study |
| 5. | Donders GG, Van Bulck B, Van de Walle P, Kaiser RR, Pohlig G, Gonser S, Graf F. Effect of lyophilized lactobacilli and 0.03 mg estriol (Gynoflor®) on vaginitis and vaginosis with disrupted vaginal microflora: a multicenter, randomized, single-blind, active-controlled pilot study. Gynecol Obstet Invest. 2010;70(4):264-72. doi: 10.1159/000314016. | There was no verification of cure, insufficient information/no clear reporting to delineate number of patients with recurrence, high drop out rate at month 4 (two-third), mixture of BV (14:14) and AV (8:10) in each arm, resulting in relatively small sample size |
| 6. | Reid G, Beuerman D, Heinemann C, Bruce AW. Probiotic Lactobacillus dose required to restore and maintain a normal vaginal flora. FEMS Immunol Med Microbiol. 2001;32(1):37-41. doi: 10.1111/j.1574-695X.2001.tb00531.x. | Did not fulfill the review's inclusion criteria of following up at least after a single menstrual cycle (14 days), however, it is possible to include the study if we take the cutoff of intervention at D14, and outcome at D42, allowing a 28-days interval |
| 7. | Reid G, Charbonneau D, Erb J, Kochanowski B, Beuerman D, Poehner R, Bruce AW. Oral use of Lactobacillus rhamnosus GR-1 and L. fermentum RC-14 significantly alters vaginal flora: randomized, placebo-controlled trial in 64 healthy women. FEMS Immunol Med Microbiol. 2003;35(2):131-4. doi: 10.1016/S0928-8244(02)00465-0. | A mixture of participants, healthy women without BV and women with BV, impossible to delineate which results belong to who |
| 8 | Anukam K, Osazuwa E, Ahonkhai I, Ngwu M, Osemene G, Bruce AW, Reid G. Augmentation of antimicrobial metronidazole therapy of bacterial vaginosis with oral probiotic Lactobacillus rhamnosus GR-1 and Lactobacillus reuteri RC-14: randomized, double-blind, placebo controlled trial. Microbes Infect. 2006;8(6):1450-4. doi: 10.1016/j.micinf.2006.01.003. | Did not fulfill the review's inclusion criteria of following up at least after a single menstrual cycle upon cure of BV (no verification of cure at baseline prior commencement of probiotics) |
| 9 | Anukam KC, Osazuwa E, Osemene GI, Ehigiagbe F, Bruce AW, Reid G. Clinical study comparing probiotic Lactobacillus GR-1 and RC-14 with metronidazole vaginal gel to treat symptomatic bacterial vaginosis. Microbes Infect. 2006;8(12-13):2772-6. doi: 10.1016/j.micinf.2006.08.008. | Did not fulfill the review's inclusion criteria of following up at least after a single menstrual cycle upon cure of BV (verification of cure at D6, followed up at D30, a max interval of only 24 days) |
| 10 | Balaghi Z, Azima S, Motamedifar M, Kaviani M, Poordast T, Zare N. The Effect of Lactofem Oral Probiotic Capsule on Lactobacilli Colonization and Some Vaginal Health Parameters. Gynecol Obstet Invest. 2020;85(3):245-251. doi: 10.1159/000506802. | The study exclusively recruited participants of Nugent score 4-6 (intermediate) |
| 11 | Mastromarino P, Macchia S, Meggiorini L, Trinchieri V, Mosca L, Perluigi M, Midulla C. Effectiveness of Lactobacillus-containing vaginal tablets in the treatment of symptomatic bacterial vaginosis. Clin Microbiol Infect. 2009;15(1):67-74. doi: 10.1111/j.1469-0691.2008.02112.x. | Did not fulfill the review's inclusion criteria of following up at least after a single menstrual cycle upon cure of BV (maximum interval was 21 days from the commencement of verum) |
| 12 | Martinez RC, Franceschini SA, Patta MC, Quintana SM, Gomes BC, De Martinis EC, Reid G. Improved cure of bacterial vaginosis with single dose of tinidazole (2 g), Lactobacillus rhamnosus GR-1, and Lactobacillus reuteri RC-14: a randomized, double-blind, placebo-controlled trial. Can J Microbiol. 2009;55(2):133-8. doi: 10.1139/w08-102. | Did not fulfill the review's inclusion criteria of following up at least after a single menstrual cycle upon cure of BV (intervention for 28 days, assessment only at D28 without any in-between or further follow up) |
| 13 | Ling Z, Liu X, Chen W, Luo Y, Yuan L, Xia Y, Nelson KE, Huang S, Zhang S, Wang Y, Yuan J, Li L, Xiang C. The restoration of the vaginal microbiota after treatment for bacterial vaginosis with metronidazole or probiotics. Microb Ecol. 2013;65(3):773-80. doi: 10.1007/s00248-012-0154-3. | Did not fulfill the review's inclusion criteria of following up at least after a single menstrual cycle upon cure of BV (cured at D5 of probiotics, however, subsequent follow up at D30 most likely did not allow enough time for one menstrual cycle to happen) |
| 14 | Hemalatha R, Mastromarino P, Ramalaxmi BA, Balakrishna NV, Sesikeran B. Effectiveness of vaginal tablets containing lactobacilli versus pH tablets on vaginal health and inflammatory cytokines: a randomized, double-blind study. Eur J Clin Microbiol Infect Dis. 2012;31(11):3097-105. doi: 10.1007/s10096-012-1671-1. | Did not fulfill the review's inclusion criteria of following up at least after a single menstrual cycle upon cure of BV (whole trial only lasted 9 days) |
| 15 | Petricevic L, Witt A. The role of Lactobacillus casei rhamnosus Lcr35 in restoring the normal vaginal flora after antibiotic treatment of bacterial vaginosis. BJOG. 2008;115(11):1369-74. doi: 10.1111/j.1471-0528.2008.01882.x. | Did not fulfill the review's inclusion criteria of following up at least after a single menstrual cycle upon cure of BV (verification of cure was done at day 30 post-completion of treatment, with no further follow up) |
| 16 | De Gregorio PR, Maldonado NC, Pingitore EV, Terraf MCL, Tomás MSJ, de Ruiz CS, Santos V, Wiese B, Bru E, Paiz MC, Reina MF, Schujman DE, Nader-Macías MEF. Intravaginal administration of gelatine capsules containing freeze-dried autochthonous lactobacilli: a double-blind, randomised clinical trial of safety. Benef Microbes. 2020;11(1):5-17. doi: 10.3920/BM2019.0081. | Did not fulfill the review's inclusion criteria of following up at least after a single menstrual cycle upon cure of BV (f/u to within 7 days after completion of intervention; confusing article, abstract: 7 applicators each for a day, and f/u 3-10days after completion of intervention; methods: 7 applicators to be applied 7th to 10th day of menstrual cycle, f/u within 7 days after completion of intervention) |
| 17 | Eriksson K, Carlsson B, Forsum U, Larsson PG. A double-blind treatment study of bacterial vaginosis with normal vaginal lactobacilli after an open treatment with vaginal clindamycin ovules. Acta Derm Venereol. 2005;85(1):42-6. doi: 10.1080/00015550410022249. PMID: 15848990. | Insufficient information: the paper focused on cure rate. although there was follow-up up till 2nd menstrual cycle (self-collected swab) and clindamycin was given 3 days for treating the BV, the "results" were poorly reported and only cure rate for intermediate BV (Nugent 4-6) were reported for the swab after the 2nd menstrual cycle. |
| 18 | van de Wijgert JHHM, Verwijs MC, Agaba SK, Bronowski C, Mwambarangwe L, Uwineza M, Lievens E, Nivoliez A, Ravel J, Darby AC. Intermittent Lactobacilli-containing Vaginal Probiotic or Metronidazole Use to Prevent Bacterial Vaginosis Recurrence: A Pilot Study Incorporating Microscopy and Sequencing. Sci Rep. 2020;10(1):3884. doi: 10.1038/s41598-020-60671-6. | Small sample size; 10 in non-probiotics, 11 in probiotics, calculated based on Nugent score as shown in Table 3 of article. Besides, exclusion of Nugent positive but modified Amsel negative patients. Usage of modified Amsel instead of conventional Amsel |
| 19 | Russo R, Karadja E, De Seta F. Evidence-based mixture containing Lactobacillus strains and lactoferrin to prevent recurrent bacterial vaginosis: a double blind, placebo controlled, randomised clinical trial. Benef Microbes. 2019;10(1):19-26. doi: 10.3920/BM2018.0075. | Intervention were lactobacilli combined with latoferrin. Hence, could not delineate the beneficial effects were attributable to which component of the intervention |
| 20 | Di Pierro F, Catacchio V, Candidi C, Zerbinati N, Alfonso R. Rhatany-based preparation in vulvovaginitis and vaginosis. Gazzetta Medica Italiana Archivio per le Scienze Mediche 2009;168(6):339-46 | Did not fulfill the review's inclusion criteria of following up at least after a single menstrual cycle upon cure of BV (swab taken 17 days after commencement of probiotics), intervention contained both lactic acid and probiotic) |
| 21 | Drago L, De Vecchi E, Nicola L, Zucchetti E, Gismondo MR, Vicariotto F. Activity of a Lactobacillus acidophilus-based douche for the treatment of bacterial vaginosis. J Altern Complement Med. 2007;13(4):435-8. doi: 10.1089/acm.2006.6040. | All patients (n=40) received probiotics, BV was treated with probiotics instead of standard antibiotics, did not fulfill the review's inclusion criteria of following up at least after a single menstrual cycle upon cure of BV (14 days). |
| 22 | Ziyadi S, Homayouni A, Mohammad-Alizadeh-Charandabi S, Bastani, P. Probiotics and Usage in Bacterial Vaginosis. In: Probiotics, Prebiotics, and Synbiotics. 2019: 655–659. doi:10.1016/b978-0-12-802189-7.00049-6 | Administration of probiotic in the form of yoghurt |
| 23 | Thulkar J, Kriplani A, Agarwal N. Probiotic and metronidazole treatment for recurrent bacterial vaginosis. Int J Gynaecol Obstet. 2010;108(3):251-2. doi: 10.1016/j.ijgo.2009.09.029. Epub 2009 Nov 25. PMID: 19939382. | Small sample size (n=8) |
| 24 | Tena Gonzalez JA, San Juan P, Herraiz Esteban N, de Castellar Sanso R, Tort Sanchez G. Boric acid and probiotics in bacterial and fungal vulvovaginitis as an effective therapeutic option. Progresos de obstetricia y ginecologia, 2019, 62(3), 221‐229 \| https://doi.org/10.20960/j.pog.00195 | Small sample size (n=6) out of 48. 42 others have candida vulvovaginitis; study also included suspected cases instead of exclusively diagnosed case; intervention arm received both probiotic and boric acid instead of probiotic exclusively. |
| 25 | Lin T-C, Hsu I-L, Tsai W-H, Chu Y-C, Kuan L-C, Huang M-S, Yeh W-L, Chen Y-H, Hsu S-J, Chang W-W. Improvement of Bacterial Vaginosis by Oral *Lactobacillus* Supplement: A Randomized, Double-Blinded Trial. Applied Sciences. 2021; 11(3):902. https://doi.org/10.3390/app11030902 | Fulfilled the operational definition of recurrence, however, both arms also receiving probiotics |
| 26 | Ratna Sudha M, Yelikar KA, Deshpande S. Clinical Study of Bacillus coagulans Unique IS-2 (ATCC PTA-11748) in the Treatment of Patients with Bacterial Vaginosis. Indian J Microbiol. 2012;52(3):396-9. doi: 10.1007/s12088-011-0233-z. | There was no verification of cure, authors however, claimed minimization of recurrence |
| 27 | Verdenelli MC, Cecchini C, Coman MM, Silvi S, Orpianesi C, Coata G, Cresci A, Di Renzo GC. Impact of Probiotic SYNBIO(®) Administered by Vaginal Suppositories in Promoting Vaginal Health of Apparently Healthy Women. Curr Microbiol. 2016;73(4):483-90. doi: 10.1007/s00284-016-1085-x. | Exclusively recruited normal and intermediate Nugent score women; did not fulfill the review's inclusion criteria of following up at least after a single menstrual cycle upon cure of BV (longest interval to f/u was 28 days) |
| 28 | Sudha MR, Maurya AK. Effect of oral supplementation of the probiotic capsule UB-01BV in the treatment of patients with bacterial vaginosis. Beneficial Microbes. 2012;3(2):151-155. DOI: 10.3920/bm2011.0054. | Did not fulfill the review's inclusion criteria of following up at least after a single menstrual cycle upon cure of BV (longest interval of f/u was 15 days) |
| 29 | Harasim-Dylak, A.; Roguska, M.; Ma´zdziarz, A. Effectiveness of Trivagin in restoring and maintaining normal vaginal ecosystem in women treated for recurrent bacterial vaginosis. Curr. Gynecol. Oncol. 2011, 9, 245–252 | Probiotic was used as treatment, focused on efficacy rather than recurrence |
| 30 | Marcotte H, Larsson PG, Andersen KK, Zuo F, Mikkelsen LS, Brandsborg E, Gray G, Laher F, Otwombe K. An exploratory pilot study evaluating the supplementation of standard antibiotic therapy with probiotic lactobacilli in south African women with bacterial vaginosis. BMC Infect Dis. 2019;19(1):824. doi: 10.1186/s12879-019-4425-1. | Small sample size on recurrence (n=5 in intervention; n=4 in controlled) |
| 31 | Tafazzoli HH, Amiraliakbari S, Afrakhteh M, AlaviMajd H, Nouraei S, et al. (2014) Comparison of Metronidazole versus a Combination of Metronidazole plus Probiotics in the Treatment of Bacterial Vaginosis. J Womens Health, Issues Care 3:3. doi:10.4172/2325-9795.1000146 | Did not fulfill the review's inclusion criteria of following up at least after a single menstrual cycle upon cure of BV (whole trial lasted 4 weeks); no controlled group in which both group received probiotics of different strains) |
| 32 | Larsson PG, Brandsborg E, Forsum U, Pendharkar S, Andersen KK, Nasic S, Hammarström L, Marcotte H. Extended antimicrobial treatment of bacterial vaginosis combined with human lactobacilli to find the best treatment and minimize the risk of relapses. BMC Infect Dis. 2011;11:223. doi: 10.1186/1471-2334-11-223. | No controlled group; prospective, cross-sectional study |
| 33 | Pino A, Rapisarda AMC, Vitale SG, Cianci S, Caggia C, Randazzo CL, Cianci A. A clinical pilot study on the effect of the probiotic Lacticaseibacillus rhamnosus TOM 22.8 strain in women with vaginal dysbiosis. Sci Rep. 2021;11(1):2592. doi: 10.1038/s41598-021-81931-z. | There was no verification of cure in controlled group (refer to Table 5) |
| 34 | Ratna Sudha M, Yelikar KA, Deshpande S. Clinical Study of Bacillus coagulans Unique IS-2 (ATCC PTA-11748) in the Treatment of Patients with Bacterial Vaginosis. Indian J Microbiol. 2012;52(3):396-9. doi: 10.1007/s12088-011-0233-z. | Did not fulfill the review's inclusion criteria of following up at least after a single menstrual cycle upon cure of BV (there no verification of cure although the trial was for 3 months, thus it's fair to say could only delineate efficacy at 3-months interval in treating BV) |
| 35 | Reid G, Burton J, Hammond JA, Bruce AW. Nucleic acid-based diagnosis of bacterial vaginosis and improved management using probiotic lactobacilli. J Med Food;7(2):223-8. doi: 10.1089/1096620041224166. | Small sample size (n=8 in each arm); did not fulfill the review's inclusion criteria of following up at least after a single menstrual cycle upon cure of BV |
| 36 | Rossi A, Rossi T, Bertini M, Caccia G. The use of Lactobacillus rhamnosus in the therapy of bacterial vaginosis. Evaluation of clinical efficacy in a population of 40 women treated for 24 months. Arch Gynecol Obstet. 2010;281(6):1065-9. doi: 10.1007/s00404-009-1287-6. | No control group; more of a prospective cohort study |
| 37 | Bodean O, Munteanu O, Cirstoiu C, Secara D, Cirstoiu M. Probiotics--a helpful additional therapy for bacterial vaginosis. J Med Life. 2013;6(4):434-6. | Poor quality study: under-reported methodology, unclear reporting of results (contradicting figure and vague in-text), absence of inferential analysis |
| 38 | Pendharkar, S., Brandsborg, E., Hammarström, L. *et al.* Vaginal colonisation by probiotic lactobacilli and clinical outcome in women conventionally treated for bacterial vaginosis and yeast infection. BMC Infect Dis **15**, 255 (2015). https://doi.org/10.1186/s12879-015-0971-3 | No control group, refer Figure 3. small sample size (n=10 in each arm) |
| 39 | Bertini M. Is Lactobacillus rhamnosus BMX 54 vaginal application a strategy to counteract bacterial vaginosis recurrences? Proceedings of 18th World Congress on Controversis in Obstetrics, Gynecology and Infertility (COGI), WienOctober 24 – 27, 2013 | Mini review paper |
| 40 | Ozkinay E, Terek MC, Yayci M, Kaiser R, Grob P, Tuncay G. The effectiveness of live lactobacilli in combination with low dose oestriol (Gynoflor) to restore the vaginal flora after treatment of vaginal infections. BJOG. 2005;112(2):234-40. doi: 10.1111/j.1471-0528.2004.00329.x. | Small sample size (total n=19 in two arms; 10;9) |
| 41 | Cianci A, Cicinelli E, De Leo V, Fruzzetti F, Massaro MG, Bulfoni A, Parazzini F, Perino A. Observational prospective study on Lactobacillus plantarum P 17630 in the prevention of vaginal infections, during and after systemic antibiotic therapy or in women with recurrent vaginal or genitourinary infections. J Obstet Gynaecol. 2018;38(5):693-696. doi: 10.1080/01443615.2017.139 9992. | Prospective, observational study (physicians and participants choose treatment) |
| 42 | Patil D, Geetha S, Raghuprasada MS, Umakant NP. Efficacy of supplementation of probiotics along with antimicrobial agents in vulvovaginal infections in a tertiary care hospital. Asian Journal of Pharmaceutical and Clinical Research. 2021;14(6): 119-122 | Small sample size (n=13 in each arm) |
| 43 | Shamsu R, Vaman J, Nirmala C. Role of probiotics in lower reproductive tract infection in women of age group 18 to 45 years. International Journal of Reproduction, Contraception, Obstetrics and Gynecology. 2017;6(2):671-681 | Small sample size (n=13 in test group; n=5 in placebo group) refer Table 8-10 |
| 44 | Recine N, Palma E, Domenici L, Giorgini M, Imperiale L, Sassu C, Musella A, Marchetti C, Muzii L, Benedetti Panici P. Restoring vaginal microbiota: biological control of bacterial vaginosis. A prospective case-control study using Lactobacillus rhamnosus BMX 54 as adjuvant treatment against bacterial vaginosis. Arch Gynecol Obstet. 2016;293(1):101-107. doi: 10.1007/s00404-015-3810-2. | Prospective case-control study (no randomization) |
| 45 | Happel AU, Singh R, Mitchev N, Mlisana K, Jaspan HB, Barnabas SL, Passmore JS. Testing the regulatory framework in South Africa - a single-blind randomized pilot trial of commercial probiotic supplementation to standard therapy in women with bacterial vaginosis. BMC Infect Dis. 2020;20(1):491. doi: 10.1186/s12879-020-05210-4. | Small sample size (n<10) |
| 46 | Hemmerling A, Harrison W, Schroeder A, Park J, Korn A, Shiboski S, Foster-Rosales A, Cohen CR. Phase 2a study assessing colonization efficiency, safety, and acceptability of Lactobacillus crispatus CTV-05 in women with bacterial vaginosis. Sex Transm Dis. 2010;37(12):745-50. doi: 10.1097/OLQ.0b013e3181e50026. | Small sample size |
| 47 | Vicariotto F, Mogna L, Del Piano M. Effectiveness of the two microorganisms Lactobacillus fermentum LF15 and Lactobacillus plantarum LP01, formulated in slow-release vaginal tablets, in women affected by bacterial vaginosis: a pilot study. J Clin Gastroenterol. 2014;48 Suppl 1:S106-12. doi: 10.1097/MCG.0000000000000226. | Small sample size |
| 48 | Heczko PB, Tomusiak A, Adamski P, Jakimiuk AJ, Stefanski G, Mikolajczyk-Chichonska A, Suda-Szczurek M, Strus M. Supplementation of standard antibiotic therapy with oral probiotics for bacterial vaginosis and aerobic vaginitis: a randomised, double-blind, placebo-controlled trial. BMC Women's Health **15**, 115 (2015). https://doi.org/10.1186/s12905-015-0246-6 | Insufficient information: reporting of 51 recurrences. However, was not able to delineate the proportion in accordance to each arm. |
| 49 | Ehrström S, Daroczy K, Rylander E, Samuelsson C, Johannesson U, Anzén B, Påhlson C. Lactic acid bacteria colonization and clinical outcome after probiotic supplementation in conventionally treated bacterial vaginosis and vulvovaginal candidiasis. Microbes Infect. 2010;12(10):691-9. doi: 10.1016/j.micinf.2010.04.010. | Mixture of vulvovaginal candidiasis and bacterial vaginosis |
| 50 | Elsharkawy I, Noureldin E, Mohamed E, Mohamed A, Abdeldayem H, Mansour SAM. Continuous versus interrupted use of vaginal probiotics plus vaginal clindamycin cream for bacterial vaginosis: a randomized controlled study. J Matern Fetal Neonatal Med. 2021;34(1):58-65. doi: 10.1080/14767058.2019.1588246. | Both arms receiving probiotics |
