# Supplementary material for: Probiotics, a promising therapy to reduce the recurrence of bacterial vaginosis in women? a systematic review and meta-analysis of randomized controlled trials
Source: Front Nutr. 2022 Sep 20;9:938838. doi: 10.3389/fnut.2022.938838 (PMC9530327; doi:10.3389/fnut.2022.938838)
Supplement: Supplementary file 2 [file Data_Sheet_2.pdf]

Intention-to-treat

Unique ID	Study ID	D1	D2	D3	D4	D5	Overall
1	Zhang Y. et al.	-	-	+	+	-	-
2	Vujic G. et al.	+	+	+	+	+	+
3	Reznichenko H. et al.	+	+	+	+	+	+
4	Ya W. et al.	+	+	+	+	+	+
5	Cohen C.R. et al.	+	+	+	+	+	+
6	Bohbot J.M. et al.	!	+	+	+	!	!
7	Bradshaw C.S. et al.	+	!	+	+	+	!
8	Marcone V. et al. (2010)	-	+	+	+	-	-
9	Marcone V. et al. (2008)	-	+	+	+	+	-
10	Larsson P.G. et al.	!	+	+	+	+	!

+	Low risk
!	Some concerns
-	High risk

D1	Randomisation process
D2	Deviations from the intended interventions
D3	Missing outcome data
D4	Measurement of the outcome
D5	Selection of the reported result
