# Supplementary material for: Probiotics, a promising therapy to reduce the recurrence of bacterial vaginosis in women? a systematic review and meta-analysis of randomized controlled trials
Source: Front Nutr. 2022 Sep 20;9:938838. doi: 10.3389/fnut.2022.938838 (PMC9530327; doi:10.3389/fnut.2022.938838)
Supplement: Supplementary file 3 [file Data_Sheet_3.docx]

| **Index** | **Author** | **Publication year** | **Study design** | **Country** | **Cohort size (including drop out)** | **Population (inclusion criteria and method of diagnosis)** | **Intervention** | **Route of administration** | **Comparator** | **Interval of visiting** | **Primary outcome** | **Results** | **Secondary outcome** | **Results** | **Adverse effects** | **Risk of bias** |
| --- | --- | --- | --- | --- | --- | --- | --- | --- | --- | --- | --- | --- | --- | --- | --- | --- |
| 1 | Y. Zhang Y. et al. | 2021 | Single-centre, open-label, double arms, parallel randomized controlled trial | China | 126 | Women with abnormal vaginal discharge symptoms, age range 18-65, premenopausal with history of sexual activity, diagnosed BV using Nugent’s Gram stain score of >6 | L. rhamnosus GR-1 and L. reuteri RC-14 ((≥1x10^9^ CFU per day,  for 30 days, in adjunct to vaginally-administered metronidazole suppositories (n=63) | Oral: sachet | vaginally-administered metronidazole suppositories only (n=63) | Day0, 30 and 90 days after starting treatment | Cure rate (% of participants without BV i.e. Nugent score<7) during follow up, mean difference between two/multiple groups (Wilcoxon-Test/Kruskal test, CI 99%), | Experimental: 30/52, 57.69% cured at D30, 36.54% at D90; Control: 28/47, 59.57% cured at D30, 48.94% at D90  However, no significant difference OR 0.97 (95% CI,0.70-1.35, p=0.04 at D30, OR0.75(95% CI,0.47-1.19,p=0.213 at D90). | Recurrence rate, adverse effects  Quantification on abundance of vaginal and faecal microbiota  Occurrence ratio | Experimental: 11/30 recurrent at D90  Control: 5/28 recurrent at D90  No significant difference in abundance of any genera; vaginal, p=0.75 at D30, p=0.11 at D90, no p reported for faecal. | Self reported  a) 11 reported vaginal itching/burning (6 in metronidazole group and 5 in probiotic group ;  b)  6 Had mild vaginal bleeding (3 cases in each group);  c) 3 exhibited abnormal vaginal discharge (2 in probiotics and 1 in metronidazole group);  d) 2 cases experienced mild lower abdominal pain in probiotics group;  e) 1 haematochezia in probiotics group;  f) 1 opsomenorrhea in metronidazole group;  g) 1 abdominal distention in probiotics group;    h) 1 frequent urination in metronidazole group | High |
| 2 | G. Vujic et al. | 2012 | Multicentric, double-blinded, double arms, parallel, randomized placebo-controlled trial | Croatia | 544 | Women older than 18-year-old, diagnosed with vaginal infection (BV, candidiasis, trichomoniasis, or combination of these)  Diagnosis of BV based on either Amsel criteria or Nugent scoring system | 2 capsules of “Lactogyn” per day for 6/52 (each capsule containing >109 CFU Lactobacillus rhamnosus GR-1 and L. reuteri RC-14) (n=395) | Oral:capsules | Identical-looking placebo capsules (n=149) | W6, W12 from commencement of study | Rate of restitution after 6/52 | Experimental: 243/395 vs Controlled: 40/149 | Rate of BV recurrence 6 weeks after completion of intervention (indicated by disruption of restored normal vaginal microbiota) | Experimental: -1/93 (none got recurrence, in fact got one resolved BV case 6 weeks after completion of intervention) vs Controlled: 3/36 | No side effects experienced. | Low |
| 3 | H. Reznichenko et al. | 2020 | Multicentre, double-blinded, double arms, parallel, randomized placebo-controlled trial | Ukraine | 172 | Premenopausal women aged 18-45, had recent BV cured with metronidazole and proven clear of BV for at least 48 hours prior to commencement of intervention.  BV recurrence defined as presence of 3 out of 4 Amsel criteria and one or combination of self-reported symptoms typical to BV. | Test dietary supplement (TDS) combining L. crispatus LMG S-29995, L. brevis and L. acidophilus in proportion of 60%, 20%, 20% respectively, totalling to dose of 5.4 billion, taken BD for first 7 days, OD from D8 to D120 post meals (n=86) | Oral: capsule | Similar TDS- maltodextrin(identical look, smell and consistency) without lactobacilli, consumed in the same manner (n=86) | D-9, D0, D60, D120 | % of BV recurrences during 16 weeks of intervention | Experimental: D-9,82/82 BV; D0,0/82 BV; D60,5/82 recurred; D120,15/82 recurred  Controlled: D-9,84/84 BV; D0,0/84 BV; D60,16/84 recurred; D120,27/84 recurred  p<0.05 for both D60 and D120 | Nugent scores at baseline visit, D60 and D120  Time to recurrence  Rate of survival without BV | Baseline: 0 with score 7-10 in both groups; D60: 2/82 in verum,12/84 in placebo with score 7-10 (p<0.01); D120:13/82 in verum,19/84 in placebo with score 7-10  There was significant difference of time to recurrence between verum group (mean(SD);97.3(26.7)) versus placebo (mean(SD);74.7(27.7)), (p=0.01)  Verum group survived longer without BV than its counterpart | 1/82 experienced abdominal pain, 1/84 had constipation in test group; 6 intolerance events were documented by one for every  item (bloating, abdominal pain, nausea, distension, pruritus, constipation) in placebo group  The effects were mild or moderate, with  no consistent relationship attributed to the TDS | Low |
| 4 | Ya et al. | 2010 | Single centre, double-blinded, double arms, parallel randomized placebo-controlled trial | China | 120 | Chinese women 18-55-year-old, currently free of BV but had 2 or more episode of BV (recurrent) in previous year, no antibiotic treatment within 1 week of study commencement, willing to refrain from other intravaginal products  Amsel criteria used to detect recurrence | Daily 1 capsule containing 8 billion colony-forming units of lactobacilli- L. rhamnosus (6.8 billion), L. acidophilic (0.4 billion) and Strep. Thermophilus (0.8 billion) for 7 days on, 7 days off, and 7 days on (n=58) | Vaginal: capsule | 1 placebo capsule with identical appearance, smell, texture and contained only lactose (n=62) | Visits at 30 and 60 days after treatment  Follow up via telephone at 11 months | Recurrence rate of BV | Experimental: 15.8% (9/57) vs Placebo: 45.0% (27/60), OR:0.23, p<0.001) through 2 months | G. vaginalis incidence through 2 months  Incidence rate of BV and G. vaginalis between 2-11 months | Experimental: 3.5% (2/57) vs Controlled: 18.3% (11/60), OR:0.16, p=0.02)  BV Incidence:  Experimental: 10.6% vs Placebo: 27.7%, OR 0.31, p=0.04)  G. vaginalis:  Experimental: 0% vs Placebo: 64%, p=0.08 | Aside from vaginal discharge and malodor, no adverse events were documented in both groups. | Low |
| 5 | Cohen et al. (the only study that does sensitivity analyses) | 2020 | Multicentric, double-blinded, double arms, parallel randomized placebo-controlled trial with convenient sampling | USA | 228 | Women 18-45 years old, diagnosed with BV (using Amsel criteria ¾ and Nugent score 4-10), and completed a 5-days course of vaginal metronidazole gel. | Lactin V (2 billion CFU/dose of crispatus CTV-05) for 11 weeks (4 consecutive daily dose for 1^st^ week, followed by twice-weekly doses for 10 weeks) (n=152) | Vaginal: Vaginal applicators | Placebo of same inactive formulation but without L. crispatus CTV-05 for same duration and manner (n=76) | 4, 8, 12, 24 weeks after enrolment | % of BV recurrence at W12 | 30%(46/152) in experimental vs 45%(34/76) in placebo (RR 0.66, p=0.01) | % of recurrence at W24  % of AE | 59/152 in experimental group vs 41/76 in placebo group  The percentage of participants experiencing adverse events did not significantly differ between probiotics (132 participants (87%)) and the placebo group (60 participants (79%)  Risk ratio: 1.11 (95% CI:0.98, 1.33), p=0.12 | Across the two treatment groups, the incidence and severity of  local and systemic adverse events were similar. The most  common local adverse events were abnormal  vaginal discharge, abnormal vaginal odor,  external genital irritation, and genital itching; and the most common systemic  adverse events were abdominal pain or  cramps, headache, and frequent urination.  None of the severe adverse events were  classified as being related to Lactin-V or placebo | Low |
| 6 | Bohbot et al. | 2018 | Prospective, multicentric, double blinded, double arms, parallel randomized placebo-controlled trial | France | 98 | 18 years and above, had at least 2 documented episode of BV within previous year, and currently having BV diagnosed with BV using 3 Amsel criteria, prescribed oral 500mg metronidazole BD for 7 days | 1 billion CFU/gram of L. crispatus IP 174178 in a vaginal capsule, taken daily for 14 days for 2 menstrual cycles, revisited and another 14-day course of same treatment for next two menstrual cycles (n=50) | Vaginal: capsule | Placebo vaginal capsule (n=48) | Pre-enrolment (visit 1), D0 (visit 2, verification of BV cured), D56 (visit 3), D112 (visit4), D196 (visit 5) | Number of patients with recurrence at visit 4 | 16/39 in placebo group vs 8/39 in intervention group, (p=0.0497, CI:90%) | Time for first recurrence btn V2 and V4, as well as V4 and V5  Frequency of at least a recurrence btn V2 and V4, as well as btn V4 and V5  AEs at visit 5  Overall safety at Visit 4  Compliance  Average number of recurrences per patient btn V0 and V5 | Recurrence rate:  Probiotics: 8/39 (20.5%)  Placebo: 16/39 (41.0%)  Relative risk for recurrence:  0.50 (95% CI:0.24,1.03) | 34/48 (70.8%) in placebo and 31/50(62%)  in test group that experienced at least 1 adverse event (p=0.3550).  However, only 2/48 (vulvovaginal pruritus and leucorrhea) in placebo group and  1/98 in probiotics (oral consumption of probiotics) are deemed due to interventions  5 discontinued treatment due to AE: two for metrorrhagia (one in each treatment group), one for diarrhoea (L. crispatus IP 174178 group) and two for genital mycosis (one in each treatment group) | Some concerns |
| 7 | Bradshaw et al. (outcome assessor was participants using self-collected vaginal smears) | 2012 | Single centre, double-blinded, triple arms, parallel, randomized placebo-controlled trial | Australia | 450 | 18-50 year old, with symptomatic BV, diagnosed with either Nugent score 7-10 OR Nugent score 4-10 and 3 or more Amsel’s criteria, received 400mg BD oral metronidazole for 7 days, women with genital infections were also included but treated | Arm 1: vaginal 2% clindamycin cream (in the form of applicators) for 7 nights (n=150)  Arm 2: vaginal pessary containing at least 10^7 CFU of live L.acidophilus KS400, 0.03mg oestriol and excipients for 12 nights (n=150) | Vaginal: pessary | Arm 3: Identical-appearance placebo, containing only excipients for 12 nights (n=150) | 1, 2, 3 and 6 months following the intervention | Recurrence of BV within 6 months (assessment at 1, 2, 3 and 6 months following the intervention)  Hazard ratios | M1 recurrence rate; cumulative M6 recurrence rate:  Arm 1 (5/140) vs Arm 2 (9/133) vs Arm 3 (13/135), p=0.13; Arm 1 (42/140) vs Arm 2 (37/133) vs Arm 3 (36/135), p=0.82  HR at M1; HR M6:  Arm 2 vs Arm 3 at M1: 0.75; Arm 2 vs Arm 3 at M6: 1.03 | Recurrence of abnormal vaginal flora, defined as Nugent 4-10  Hazard ratios | i) Vaginal itchiness/soreness: 41/130 (32%) in clindamycin arm; 43/121 (36%) in probiotics arm; 39/127 in placebo arm), p =0.82  ii) Nausea: 9/126 (7%) in clindamycin arm; 5/118 (4%) in probiotics arm; 16/128 (13%) in placebo arm; p=0.06  iii) Abdominal pain: 1/140 (0.7%) in clindamycin arm; 1/133 (0.8%) in probiotics arm; 2/135 (1.6%) in placebo arm; p>0.999  iv( Headache: 5/140 (3.7%) in clindamycin arm; 1/133 (0.8%) in probiotics arm; 1/135 (0.8%) in placebo arm; p=>0.999  v) Increased vaginal discharge: 0 (0%) in clindamycin arm; 6/133 (4.7%) in probiotics arm; 1/135 (0.8%) in placebo arm; p=0.006 | Self-reported by probiotic group:  43/121 vaginal itch/soreness, 5/118 nausea, 1/133 vomiting, 1/13 abdominal pain, 1/133 headache, 3/133 metallic taste, 1/133 body rash, 6/133 increased vaginal discharge, 2/133 vaginal discharge containing medication, 2/133 UTI.  Authors did not comment whether the AEs are related to the treatment. | Some concerns |
| 8 | V. Marcone et al. | 2010 | single centric, single-blinded, double arms, parallel, randomized controlled trial with convenient sampling | Italy | 49 | Women between the age of 18-45.  BV diagnosed as meetingALL of Amsel criteria | Twice daily dose of 500mg oral metronidazole for 7 days, followed by once-weekly vaginal application of 40mg of L. rhamnosus for 6 months (n=24) | Vaginal: capsule | Twice daily dose of 500mg oral metronidazole for 7 days (n=25) | 30,90,180,270,360 days after first diagnosis | % of patients with balanced vaginal ecosystem | The % for D30, D90, D180, D270 and D360, respectively were as follow:  91%, 83%, 74%, 74%, 69% (controlled group); 96%, 96%, 96%, 91%, 91% (intervention group) | Vaginosis-free at each follow up | D90:  Verum group 0/22 recurrence at D90 vs Controlled group 2/21 recurrent | Not studied. | High |
| 9 | V. Marcone et al. | 2008 | single centric, single-blinded, double arms, parallel, randomized controlled trial | Italy | 84 | Women of childbearing age (18-40) with BV, defined as meeting all of  Amsel’s criteria, had no antimicrobial therapy for conditions  other than BV during the study. | Oral metronidazole 500mg BD for 7 days, followed by 1 vaginal tablet containing 40mg i.e. >40000 CFU L. rhamnosus once a week at bedtime for 2 months 1 week after last antibiotic administration (n=42) | Vaginal: tablet | Twice daily oral metronidazole 500mg for 7 days (n=42) | 30, 90, 180 days after end of metronidazole treatment | % of remission at follow up D30 | 37/42 (88%) in test group; 34/42 (81%) in controlled group | Recurrence at D90  Recurrence at D180 | 0/37 in verum group vs 4/34 in controlled group at D90  2/37 in verum group vs 6/34 in controlled group at D180 | Not studied | High |
| 10 | P.G. Larsson | 2008 | Double-blinded, randomized placebo-controlled trial with convenient sampling | Norway | 100 | Women with BV diagnosed using Amsel criteria | vaginal clindamycin therapy followed by vaginal gelatine capsules containing 1 hundred million CFU/capsule  freeze-dried L. gasseri and 1 hundred million CFU/capsule L. rhamnosus for 10 days during 3 menstrual cycles (n=50) | Vaginal: gelatine capsules | Vaginal clindamycin therapy followed by identical appearance placebo capsule taken in same manner (n=50) | Every 28 days (self swabbed monthly after menstruation for 6 months) | Cure rate | 39/50 (78%) in placebo group; 37/48 (77%) in lactobacilli group | Recurrence at end of 6-month  Lengthening of relapse | 13/37 in the lactobacilli group recurrent vs 21/39 in placebo treated women  The lactobacilli-treated women had a significantly longer time until relapse with a log rank p=0.027 | 14 (lactobacilli group) and 12 (placebo group) reported adverse events: headache, menorrhagia, haemorrhoids, influenza, bronchitis, whiplash, asthma, urinary tract infection.  Probable/certain AE: 1 in lactobacilli group due to vaginal discomfort secondary to suspected allergy | Some concerns |

**Supplementary Material 3**: Characteristics of included studies.
