# Supplementary material for: Probiotics, a promising therapy to reduce the recurrence of bacterial vaginosis in women? a systematic review and meta-analysis of randomized controlled trials
Source: Front Nutr. 2022 Sep 20;9:938838. doi: 10.3389/fnut.2022.938838 (PMC9530327; doi:10.3389/fnut.2022.938838)
Supplement: Supplementary file 4 [file Data_Sheet_4.docx]

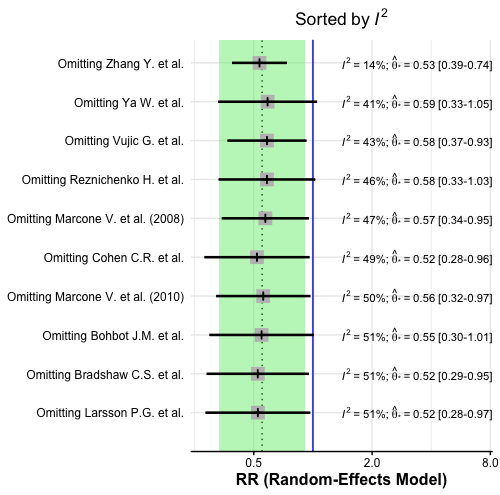


(**A**)


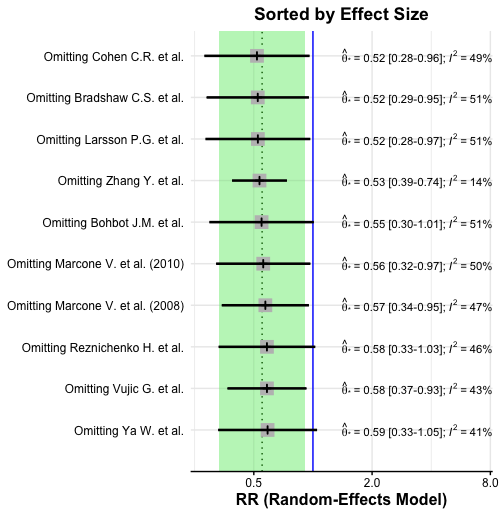


(**B**)

**Supplementary Material 4**: Influential analysis to identify any influential cases based upon (**A**) I^2^ statistics and (**B**) effect size.
