# Supplementary material for: Probiotics, a promising therapy to reduce the recurrence of bacterial vaginosis in women? a systematic review and meta-analysis of randomized controlled trials
Source: Front Nutr. 2022 Sep 20;9:938838. doi: 10.3389/fnut.2022.938838 (PMC9530327; doi:10.3389/fnut.2022.938838)
Supplement: Supplementary file 5 [file Data_Sheet_5.docx]

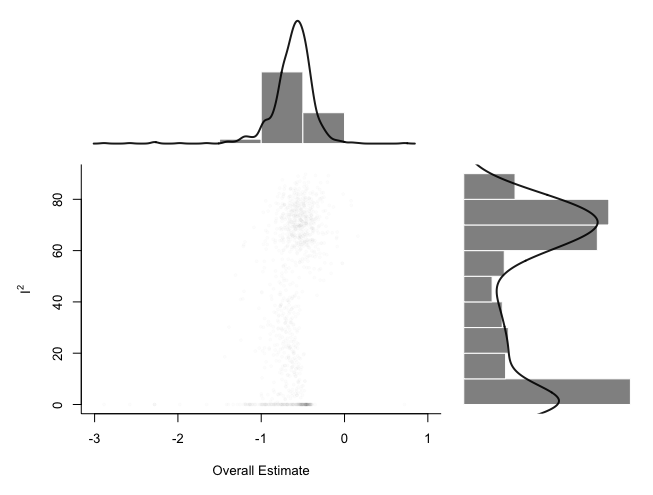
 (**A**)

(**B**)


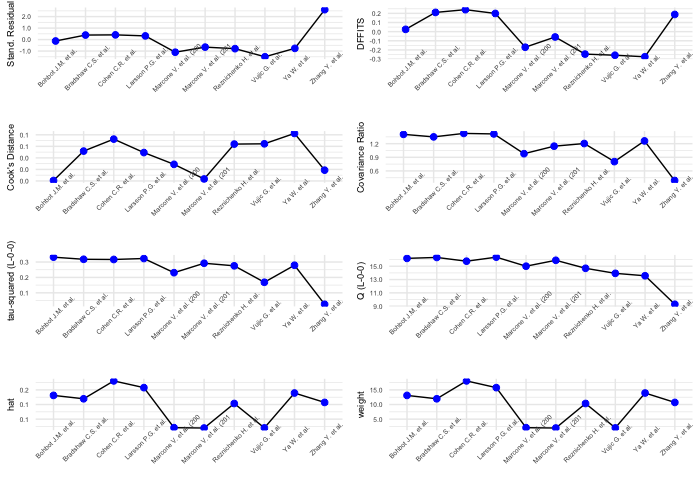


**Supplementary Material 5:** Exploration of heterogeneity pattern of studies using (**A**) GOSH plot and (**B)** GOSH diagram.
