# Supplementary material for: Probiotics, a promising therapy to reduce the recurrence of bacterial vaginosis in women? a systematic review and meta-analysis of randomized controlled trials
Source: Front Nutr. 2022 Sep 20;9:938838. doi: 10.3389/fnut.2022.938838 (PMC9530327; doi:10.3389/fnut.2022.938838)
Supplement: Supplementary file 6 [file Data_Sheet_6.docx]

**Rucker Limit:**

Review: BV Recurrence

Result of limit meta-analysis:

Random effects model RR 95%-CI z pval

Adjusted estimate 0.7359 [0.4284; 1.2640] -1.11 0.2665

Unadjusted estimate 0.5511 [0.3321; 0.9146] -2.66 0.0260

Quantifying heterogeneity:

tau^2 = 0.2460; I^2 = 45.4% [0.0%; 73.7%]; G^2 = 68.5%

Test of heterogeneity:

Q d.f. p-value

16.47 9 0.0577

Test of small-study effects:

Q-Q' d.f. p-value

3.30 1 0.0693

Test of residual heterogeneity beyond small-study effects:

Q' d.f. p-value

13.17 8 0.1061

Details on adjustment method:

- expectation (beta0)

**Supplementary Material 6:** Estimates of adjusted effect size.
