# Supplementary material for: Probiotics, a promising therapy to reduce the recurrence of bacterial vaginosis in women? a systematic review and meta-analysis of randomized controlled trials
Source: Front Nutr. 2022 Sep 20;9:938838. doi: 10.3389/fnut.2022.938838 (PMC9530327; doi:10.3389/fnut.2022.938838)
Supplement: Supplementary file 7 [file Data_Sheet_7.docx]

**
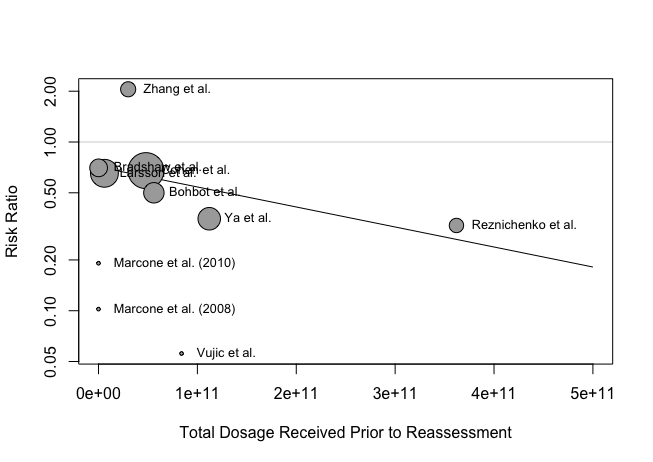
**(**A**)

(**B**)


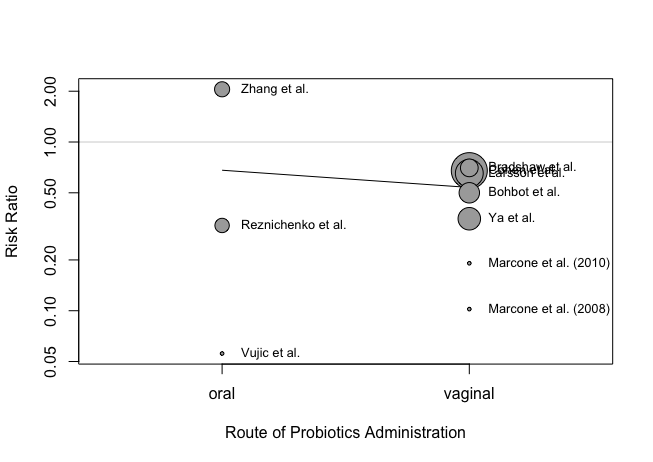


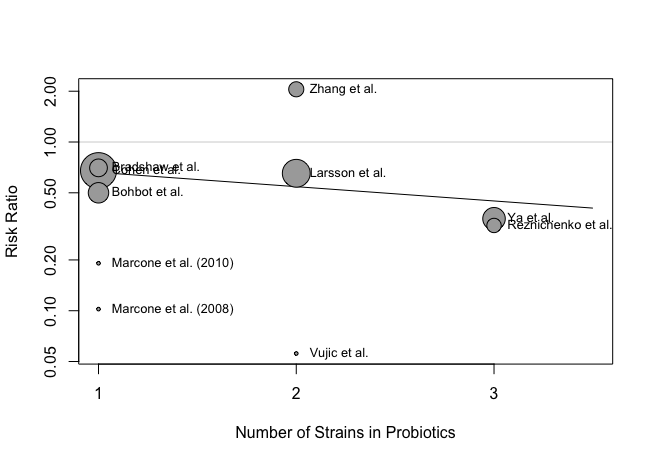
 (**C**)

(**D**)


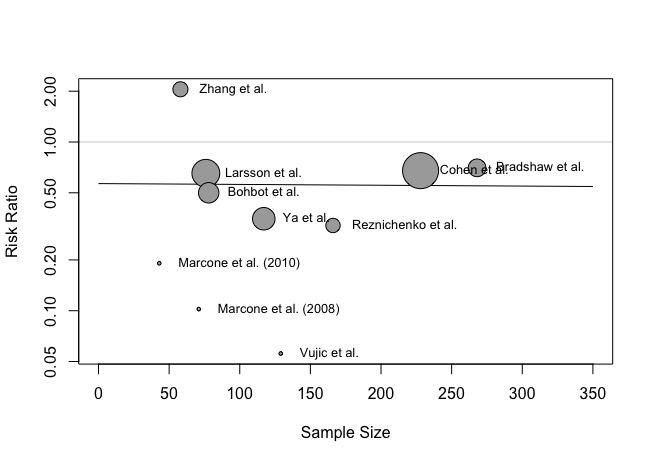


(**E**)


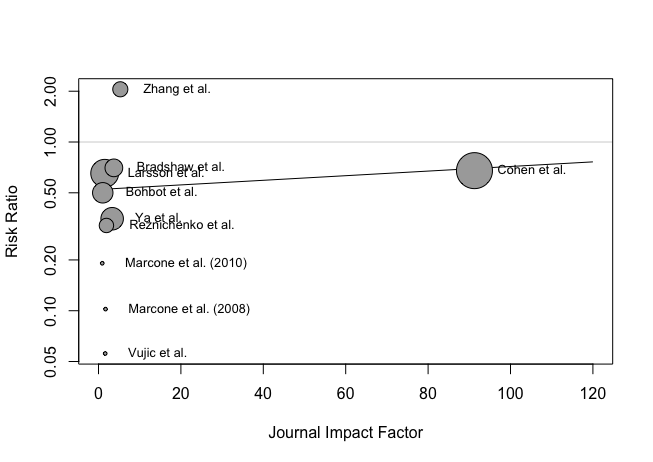


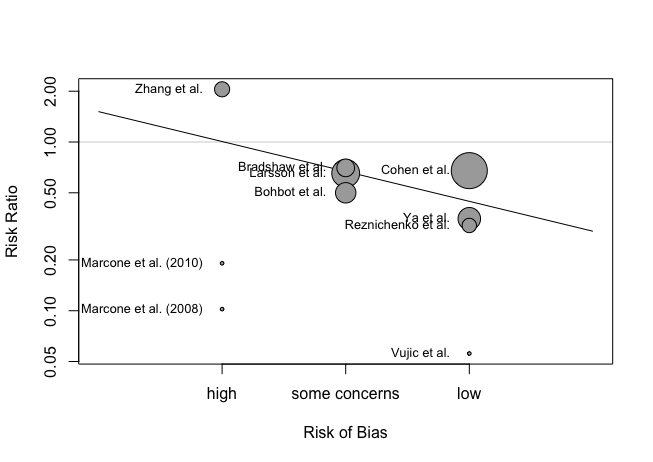


(**F**)


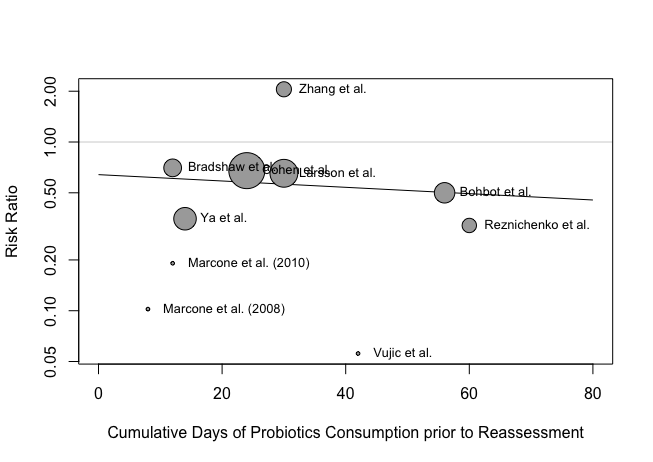
 (**G**)


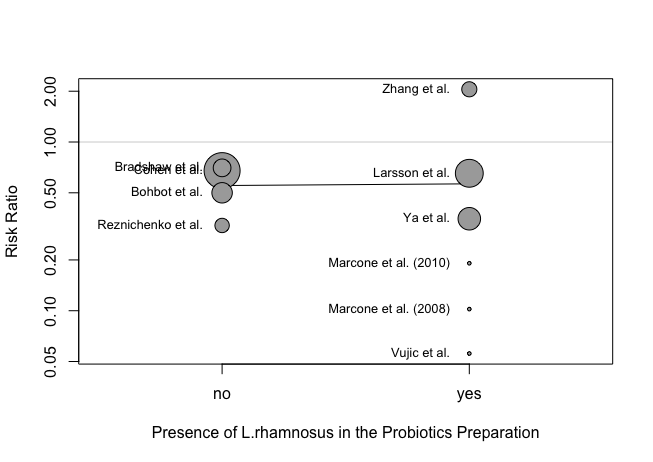
 (**H**)


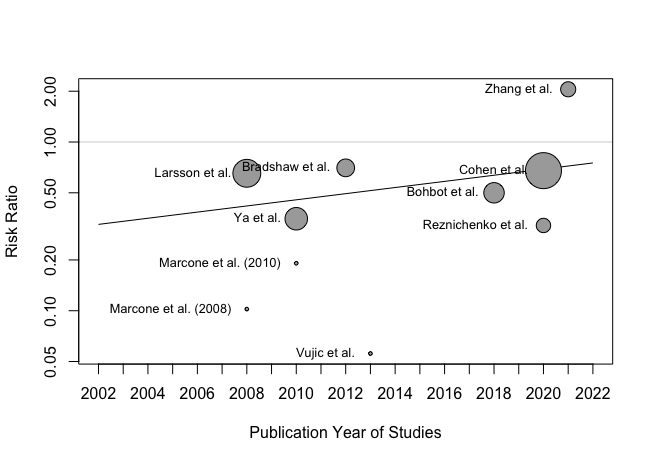
 (**I**)

**Supplementary Material 7** (**A – I**): Bubble plots on characteristics of study. (**A**) total dosage received by participants prior to reassessment of BV status (**B**) route of administration (**C**) number of strains in probiotics (**D**) sample size (**E**) impact factor (**F**) risk of bias (**G**) cumulative days of probiotics consumption (**H**) presence of Lactobacillus rhamnosus (**I**) publication year
